# The role of *DYNLT3* in breast cancer proliferation, migration, and invasion via epithelial‐to‐mesenchymal transition

**DOI:** 10.1002/cam4.6173

**Published:** 2023-06-01

**Authors:** Han Wang, Xin Chen, Yanshan Jin, Tingxian Liu, Yizuo Song, Xuejie Zhu, Xueqiong Zhu

**Affiliations:** ^1^ Department of Obstetrics and Gynecology The Second Affiliated Hospital of Wenzhou Medical University Wenzhou China; ^2^ Department of Obstetrics and Gynecology The First Affiliated Hospital of Wenzhou Medical University Wenzhou China

**Keywords:** breast cancer, *DYNLT3*, E‐cadherin, EMT, vimentin

## Abstract

**Purpose:**

*DYNLT3* is identified as an age‐related gene. Nevertheless, the specific mechanism of its carcinogenesis in breast tumor has not been clarified. This research aims to elucidate the role and the underlying molecular pathways of *DYNLT3* on breast cancer tumorigenesis.

**Methods:**

The differential expression of *DYNLT3* among breast cancer, breast fibroids, and normal tissues, as well as in various breast cancer cell lines were detected by immunohistochemical staining, real‐time quantitative reverse transcription‐PCR and Western blotting, respectively. Additionally, the role of *DYNLT3* on cell viability and proliferation were observed through cell counting kit‐8, bromodeoxyuridine, and colony formation experiments. Migratory and invasive abilities was envaulted by wound healing and Transwell methods. Apoptotic cells rate was examined by flow cytometry. Furthermore, nude mice xenograft models were established to confirm the role of *DYNLT3* in tumor formation in vivo.

**Results:**

*DYNLT3* expression was highly rising in both breast cancer tissues and cells. *DYNLT3* knockdown obviously suppressed cell growth, migration and invasion, and induced cell apoptosis in MDA‐MB‐231 and MCF‐7 breast cancer cells. The overexpression of *DYNLT3* exerted the opposite effect in MDA‐MB‐231 cells. Moreover, *DYNLT3* knockdown inhibited tumor formation in vivo. Mechanistically, an elevation of N‐cadherin and vimentin levels and a decline of E‐cadherin were observed when *DYNLT3* was upregulated, which was reversed when *DYNLT3* knockdown was performed.

**Conclusion:**

*DYNLT3* may function as a tumor‐promotor of age‐associated breast cancer, which is expected to provide experimental basis for new treatment options.

## 1 INTRODUCTION

Breast cancer has jumped to become the most prevalent type of cancer globally.^1^ In 2020, there were approximately 2,261,419 new cases diagnosed and 684,996 died worldwide.^1^ Breast cancer has the highest morbidity and mortality among female patients.^1^ Breast carcinomas possess the heterogeneity, which can be molecularly typed according to the expression patterns of estrogen receptors, progesterone receptors, and human epidermal growth factor receptor‐2 (HER‐2).^2^ In addition to surgery and chemoradiotherapy, the recent advances in endocrine and targeted therapies have made them more prominent in individual comprehensive therapy of breast cancer, which benefits the majority of patients with hormone‐dependent or HER‐2 positive breast cancer.^3^, ^4^ Unfortunately, lack of therapeutic targets often lead to a poor prognosis of triple‐negative breast cancer (TNBC).^5^ In addition, both drug resistance of advanced breast cancer and intolerance to side effects are still the bottlenecks to be solved in the treatment process.^6^

The high rate of metastasis and recurrence of breast cancer remains the causes of deaths.^7^ Even after standardized therapy, tumor metastasis occurs in approximately 20% patients with mammary carcinoma, and the risk of recurrence within 20 years is around 40%.^8^, ^9^ The capacity of tumor cells to adhere to the primary tumor is weakened by Epithelial‐to‐mesenchymal transition (EMT) and their ability to migrate and invade were enhanced, which is a prerequisite for distant tumor metastasis.^10^

Age is an important prognostic indicator for many types of tumors.^11^ It has been reported that age is not only the main hazard factor for breast cancer but also increases the mortality of female patients with breast cancer.^12^ The aging microenvironment is conducive to carcinogenesis and metastasis of tumor cells through biophysical changes in the extracellular matrix (ECM), secreted cytokines, and the immune system.^11^ The promoting effect of age in breast tumorigenesis and cancer development is caused by the successive accumulation of genetic and epigenetic alterations in mammary epithelial cells and the tumor microenvironment.^12^, ^13^, ^14^ An increase in the rigidity of the breast ECM with aging promotes the tumor cells invasion and stimulates the secretion of proliferation‐associated cytokines.^15^ Breast cancer cells interact with senescent cells that progressively accumulate unfavorable mutations, leading to tumor progression and metastasis.^15^ However, the detailed mechanisms involved in age‐induced breast cancer occurrence and progression is limited.

Cytoplasmic dynein is one of the cytoskeletal motors that drives the movement of minus‐end‐directed motors based on microtubules, responsible for the transport of vesicles, membrane‐bound organelles and mitotic movement of chromosomes.^16^, ^17^ *DYNLT3* belongs the dynein light chain gene family, which is involved in chromosome condensation and regulation of mitosis by targeting the spindle checkpoint.^18^ *DYNLT3* acted as a tumor‐promotor in ovarian carcinogenesis through impetus of cell proliferation and metastasis.^19^, ^20^ *DYNLT3* was also hypomethylated in salivary gland adenoid cystic carcinoma by genome‐wide screening.^21^ However, *DYNLT3* has been found to have tumor inhibition effect due to the reduced expression in esophageal squamous cell carcinoma by bioinformatics.^22^ Another study also found that upregulation of *DYNLT3* expression induced apoptosis and attenuated tumor metastasis in cervical cancer.^23^ Notably, recent bioinformatics research has indicated that *DYNLT3*, as one of the aging‐associated genes, was upregulated in the human mammary carcinoma.^24^ Nevertheless, studies of *DYNLT3* action in tumors are very limited, especially in terms of its function and specific mechanism on the occurrence and development of breast cancer are still indistinct.

Hence, this research was explored the role of *DYNLT3* in breast cancer. The function of *DYNLT3* in proliferation, apoptosis, migration, and invasion of tumor cells were explored by gain‐ and loss‐of function assays. In addition, whether *DYNLT3* could affect tumor growth in vivo was studied by nude mice tumor models. This research may provide new insights for developing novel strategies for aging‐associated breast cancer therapy in the future.

## 2 MATERIALS AND METHODS

### 2.1 Samples collection

A total of 15 breast cancer and 15 breast fibroids patients were enrolled in our study, diagnosed in the Second Affiliated Hospital of Wenzhou Medical University and the First Affiliated Hospital of Wenzhou Medical University from May 2019 to August 2020 who aged from 21 to 70 years old, with no medical history of mastectomy and acute infection. Breast tumor and paired para‐carcinoma tissues from 15 patients were obtained through breast cancer radical mastectomy. Breast fibroids tissues were obtained from another 15 breast fibroids patients with surgery. All samples were confirmed pathologically and none of the patients had received radiation or chemotherapy before surgery.

The ethical approval of this experiment was authorized by the ethics committee of the Second Affiliated Hospital of Wenzhou Medical University (KY‐2017‐43). The participants included in this experiment gave informed preoperative consent and relevant clinical information.

### 2.2 Immunohistochemical (IHC) staining

The tissues were treated in 4% tissue fixative and set in the automatic dehydration dehydrator for routine dehydration, transparent embedding, and paraffin embedding to make tissue wax blocks. The tissues were cut into 5 μm thick slices. After setting at 60°C for 1 h, soaking in xylene and a range of gradient ethyl alcohol for 10 min each for dewaxing, the tissue slices in sodium citrate solution were boiled in a microwave oven and then antigen repaired for 7 min on medium heat. Following treatment with 3% H_2_O_2_ and 5% bovine serum albumin (BSA), the rabbit anti‐*DYNLT3* antibody (1:100, ab121209, Abcam), the rabbit anti‐E‐cadherin antibody (1:200, 20874‐1‐AP, Proteintech), and the mouse anti‐N‐cadherin antibody (1:200, 22018‐1‐AP, Proteintech) were applied to the slides, respectively. After 16 h of incubation at 4°C, the sections were reacted with the corresponding secondary antibodies for 1 h, then stained by diaminobenzidine (DAB) for 40 s and redyed by hematoxylin for 15 s. IHC staining results were observed with an optical microscope (DMi8, Leica), each slide was randomly captured under high magnification and evaluated independently by two pathologists. The staining index was evaluated by multiplying the positive staining percentage score of tumor cells (less than 25%, 1; 26%–50%, 2; 51%–75%, 3; more than 75%, 4) by the dyeing intensity score (negative, 0; weak, 1; moderate, 2; strong, 3).

### 2.3 Cell culture

HEK‐293 T cells and the breast cancer cell lines MCF‐7, MDA‐MB‐231 (TNBC cell), HCC1937, and ZR‐75‐1 were bought from the cell bank of the Shanghai Cell Institute. HCC1937 cells were cultured in Roswell Park Memorial Institute 1640 medium (Gibco) with 10% fetal bovine serum and 1% penicillin–streptomycin (Meilunbio); other cells were cultured in Dulbecco's Modified Eagle Medium (Gibco). Particularly, MCF‐7 cells were cultured with 0.01 mg/mL insulin. All cells were cultured in a humidified incubator at 37°C with 5% CO_2_.

### 2.4 Lentivirus and cell transfection

The *DYNLT3*‐knockdown and ‐overexpressed breast cancer cell lines were established by lentivirus and cell transfection technology. The sequences of small hairpin RNA (shRNA) target *DYNLT3* were listed as follows: sh‐*DYNLT3*‐1, 5′‐CCG GTC TAT ACA GCA TCG TTT AAA TCT CGA GAT TTA AAC GAT GCT GTA TAG ATT TTT G‐3′; sh‐*DYNLT3*‐2, 5′‐CCG GTG ATG GAA CCT GTA CCG TAC TCG AGT ACG GTA CAG GTT CCA TCT TTT TG‐3′. Moreover, the human *DYNLT3* cDNA overexpression plasmid was synthesized by Changsha Youbio biosciences lnc. Target plasmid and helper plasmids (psPAX2 and pMD2.G) were mixed and transfected into HEK‐293 T with 1:1000 Lipo2000 (Invitrogen) in complete medium without penicillin–streptomycin solution. After 2 days and 3 days, the lentiviral particles generated in the cell culture medium were collected. Then, the collected lentiviral particles were filtered to infect breast cancer cells with 1:1000 polybrene (Biosharp) for 2 days. The stable *DYNLT3*‐knockdown and ‐overexpressed breast cancer cell lines were selected by 2 μg/mL puromycin (Solarbio) and validated by Western blotting.

### 2.5 Real‐time quantitative reverse transcription‐PCR (qRT‐PCR)

The expression of *DYNLT3* mRNA in breast cancer cells was detected by qRT‐PCR. A one‐step RT kit was used to prepare cDNA. Human *DYNLT3* primers, forward (5′‐AACCAGTGGACTGCAAGCAT‐3′), reverse (5′‐CCGGTTCTCCCATCTTACGG‐3′), and human GAPDH primers were used for qRT‐PCR using SYBR Green PCR Mix via the LightCycler 480 qPCR machine. A total of 45 cycles were set according to the following conditions: denature at 95°C for 10 s, anneal at 55°C for 15 s, and extend at 70°C for 1 min. The expression level of *DYNLT3* mRNA was detected following the 2^⁻ΔΔCt^ method. The relative lowest transcription level was set to “one” artificially.

### 2.6 Western blotting

The protein levels in breast cancer cells were detected by Western blotting assay as previously described.^19^ The primary antibodies consisted of the rabbit anti‐*DYNLT3* antibody (1:1000, ab121209, Abcam), the rabbit anti‐E‐cadherin antibody (1:2000, 20874‐1‐AP, Proteintech), the mouse anti‐N‐cadherin antibody (1:2000, 22,018‐1‐AP, Proteintech), the rabbit anti‐vimentin antibody (1:2000, 5741S, CST), and the mouse anti‐GAPDH antibody (1:5000, BM145, Boster). The chemiluminescence method was used to display the bands.

### 2.7 Cell counting kit‐8 (CCK‐8) assay

The cells (2 × 10^3^/well) were seeded into 96‐well plates, each group was set six parallel wells. In the following week, CCK‐8 solution (10 μL/well) was added to each well and continued to be cultured for 2 h in a cell incubator. The OD_450nm_ value was detected by a Microplate Reader (Thermo) to evaluate cell viability.

### 2.8 Colony formation assay

To further verify the role of *DYNLT3* on cell proliferation, 500 cells were seeded in six‐well plates and cultured in a 37°C, 5% CO_2_ incubator for 2 weeks until cell colonies were formed. Following that, 4% paraformaldehyde (Solarbio) and crystal violet (Beyotime) were used for 30 min and 10 min, respectively, to make the colonies visible. The number of colonies was captured and counted under a microscope (DMi8, Leica).

### 2.9 Bromodeoxyuridine (BrdU) assay

In brief, BrdU was added to the medium after the cells stuck to the well. Then the cells were treated with 4% paraformaldehyde and cleaned three times with phosphate‐buffered saline (PBS). The cells were treated with 3% BSA for 40 min, and the mouse anti‐BrdU antibody (CST, 1:500, 5292S) was added to incubate at 4°C overnight. Then, the slides were reacted with anti‐mouse antibody for 40 min. The cells were stained with DAB for 30 s and counter‐stained with hematoxylin for 20 s. Finally, the five fields of slides were randomly selected under a microscope (DMi8, Leica) for examined the presence of positive brown nuclear cells. The proliferative ability of cells was assessed by the formula: number of DAB‐stained cells per field/total cells per field.

### 2.10 Apoptosis assay

The cells were stained with Annexin V‐PE/7‐AAD (BD, Biosciences) and detected by flow cytometry to analyze the apoptotic cells proportion. The specific protocol was described in our previous study.^19^

### 2.11 Wound healing assay

After the confluence of cells approached 100%, a wound was created using a 10 μL pipette tip. Five photos of cell scratches at 0 and 24 h were taken under a microscope (DMi8, Leica), and the area of cell movement toward the wound center was calculated. The fetal bovine serum added to the medium was less than 1%.

### 2.12 Transwell assays

The cells (1 × 10^5^/well) were implanted into the upward chamber (Corning, NY, USA) and cultured in serum‐free Dulbecco's Modified Eagle Medium. A total of 700 μL of serum‐free culture medium were supplemented to each lower chamber for 1 day of culture. The cells were treated with 4% fixative for 30 min and dyed using crystal violet. Five images of each chamber were randomly acquired by an optical microscope (DMi8, Leica). The cells passing the membrane of the chamber were counted to evaluate the ability of cell migration or invasion.

### 2.13 Immunofluorescence (IF) assay

After overnight culture, cells were seeded on slides and adherent. Slides were treated with 4% formaldehyde, incubated with 0.1% Triton/PBS three times to permeabilize the cells, cleaned with PBS, and blocked with 0.1% Tween‐100 in 2% BSA for 1 h. Then the slides were incubated with primary N‐cadherin and vimentin antibodies (1:500) at 4°C for 16 h, followed by fluorescent antibodies (1:200, Invitrogen, A32744, A32740) for 40 min and stained with 4′,6‐diamidino‐2‐phenylindole (DAPI; ab104139, Abcam). Random fields were chosen to capture images with fluorescent microscopy (DMi8, Leica).

### 2.14 Tumor xenograft model

Experimental animals included in the study were approved by the Animal Ethics Committee of Wenzhou Medical University. Twenty female nude mice, aged four to 6 weeks, were randomly divided into two groups: the MCF‐7 *DYNLT3*‐knockdown group and the MCF‐7 control group. The nude mice were injected with 100 μL of PBS containing 2 × 10^6^ cancer cells subcutaneously, and each mouse was intramuscularly injected with 0.05 mg of estradiol twice a week for better tumor formation. Tumor volume was calculated as follows: length × width^2^/2. The mice were euthanized after 5 weeks of tumor growth. The resected xenografts were fixed with 4% paraformaldehyde for paraffin sections after weighting. The IHC staining was also used to investigate the expression of EMT‐related factors in tumor tissues.

### 2.15 Statistical analysis

Experimental data was analyzed with GraphPad Prism 9.0. The Kolmogorov–Smirnov test was used to determine whether the data distribution was Gaussian. For normal distribution data, the difference in the two groups and multiple groups were statistical compared through the student's *t*‐test and one‐way analysis of variance, respectively. For data that are not normally distributed, Welch's *t*‐test and the Mann–Whitney *U*‐test were conducted to analyze the difference in the two groups and multiple groups, respectively. The results were presented as the mean ± standard deviation (SD). The significance level was set at *p* < 0.05.

## 3 RESULTS

### 3.1 DYNLT3 is highly expressed in human breast cancer tissues and cells

**FIGURE 1:**
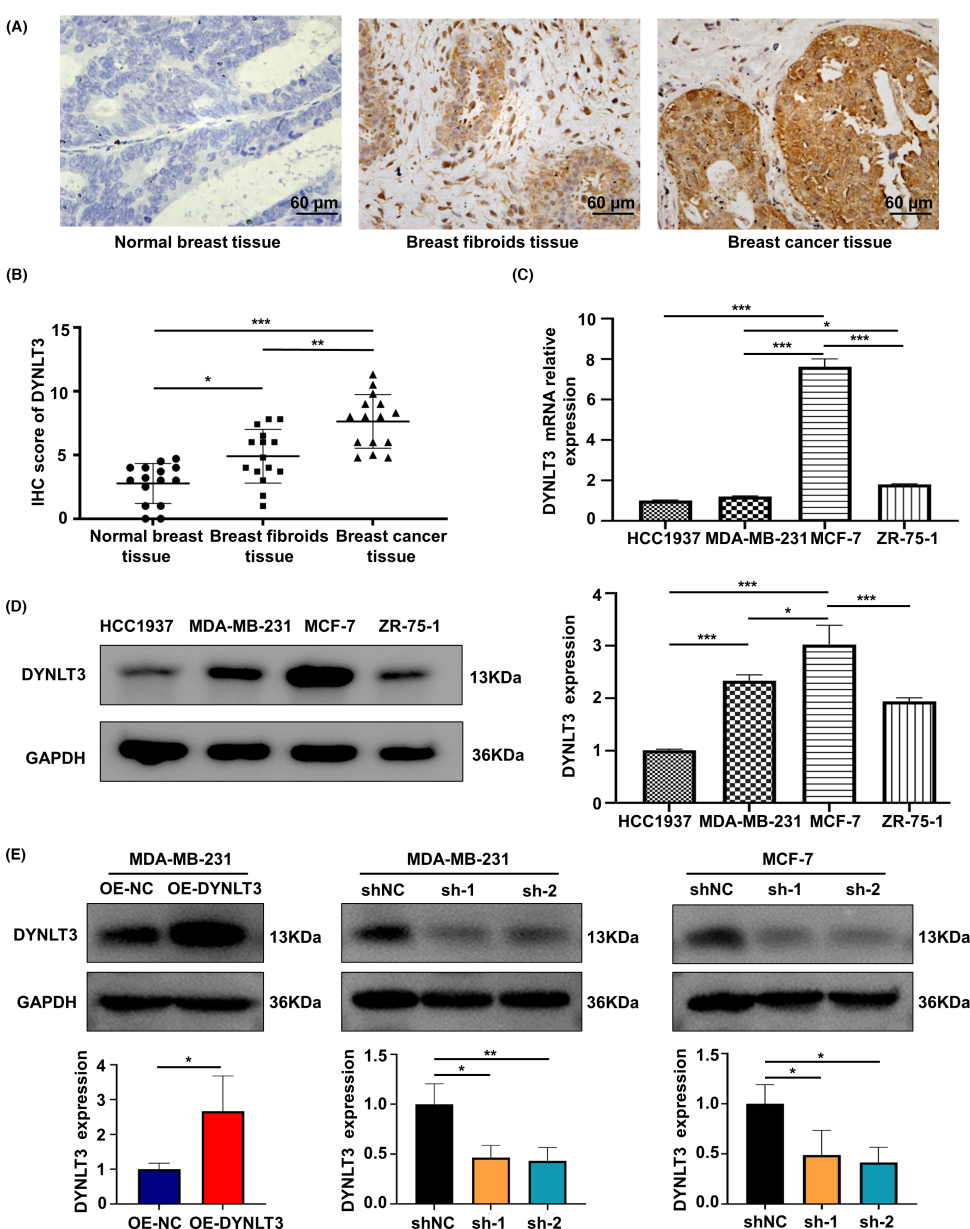
The expression level of *DYNLT3* protein in breast cancer, breast fibroids and normal breast tissues. (A,B) Immunohistochemical staining of *DYNLT3* protein and the IHC score in breast cancer, breast fibroids and normal breast tissues. (C) The expression level of *DYNLT3* mRNA in HCC1937, MDA‐MB‐231, MCF‐7, and ZR‐75‐1 breast cancer cells by qRT‐PCR. (D) Left panel: *DYNLT3* protein expression levels in HCC1937, MDA‐MB‐231, MCF‐7 and ZR‐75‐1 breast cancer cells by Western blotting. Right panel: Quantitative analysis of *DYNLT3* protein expression in breast cancer cells. (E) Construction of MDA‐MB‐231 cells with *DYNLT3* overexpression, MDA‐MB‐231 and MCF‐7 cells with *DYNLT3* knockdown. *p < 0.05, **p < 0.01, ***p < 0.001.

The 15 cases of breast cancer, 15 cases of breast fibroids, and 15 cases of normal tissues was used to detect differential expression of *DYNLT3* protein by IHC staining. The clinical information of the patients enrolled in the study were presented in Table 1. As shown in Figure 1A, the *DYNLT3* protein was located both in the cytoplasm and the nucleus. Additionally, the expression of the *DYNLT3* protein in breast fibroids and breast cancer tissues was significantly higher than that of normal breast tissues, and the expression of *DYNLT3* in breast cancer tissues was relatively highest among the three tissues. The IHC score of the *DYNLT3* protein was highest in breast cancer tissues; besides, the IHC score of breast fibroid tissue was relatively greater than that of the normal control group (Figure 1B). Taken together, it was suggested that *DYNLT3* might act as a tumor‐promoter in breast cancer.

**TABLE 1:** Clinico‐pathological information of patients with breast cancer.

Variable	No. of patients
Age	
<60	10
≥60	5
T stage	
T1	7
T2	6
T3–4	2
Lymph node metastasis	
No	9
Yes	6
AJCC TNM staging	
I–II	12
III–IV	3
Histology grade	
I (high differentiation)	2
II (moderate differentiation)	10
III (poor differentiation)	3
Pathological type	
Non special type	13 (Invasive ductal carcinoma)
Special type	2 (one case of mucinous adenocarcinoma and one case of tubular carcinoma)
ER status	
Negative	3
Positive	12
HER‐2 status	
Negative	9
Positive	6
PR status	
Negative	4
Positive	11

Abbreviations: ER, estrogen receptor; HER‐2, human epidermal growth factor receptor 2; PR, progesterone receptor.

The transcription and translation levels of *DYNLT3* among the four breast cancer cell lines were also detected by qRT‐PCR (Figure 1C) and Western blotting (Figure 1D). Both the *DYNLT3* mRNA and protein level in MCF‐7 cells were the highest among these cell lines tested. The expression of *DYNLT3* protein in MDA‐MB‐231 cells was second only to that of MCF‐7 cells. The differential expression of the *DYNLT3* protein between MDA‐MB‐231 cells and ZR‐75‐1 cells was not statistically significant. Therefore, MCF‐7 was selected for the construction of a cell model to knockdown the expression of *DYNLT3*, while MDA‐MB‐231 was selected to construct both overexpression and knockdown *DYNLT3* expression cell models.

As shown in Figure 1E, the expression of *DYNLT3* was dramatically elevated in MDA‐MB‐231 cells transfected with a lentivirus overexpressing *DYNLT3* compared with the MDA‐MB‐231 cells transfected with control lentivirus. Furthermore, transfection of puro‐sh*DYNLT3* lentivirus significantly downregulated the expression of *DYNLT3* in MDA‐MB‐231 and MCF‐7 cells when compared to the corresponding cells transfected with puro‐shNC control lentivirus. The Western blotting results confirmed that *DYNLT3*‐knockdown MDA‐MB‐231 and MCF‐7 cells, as well as *DYNLT3*‐overexpressed MDA‐MB‐231 cells were successfully constructed.

### 3.2 DYNLT3 enhances proliferation of breast cancer cells

**FIGURE 2:**
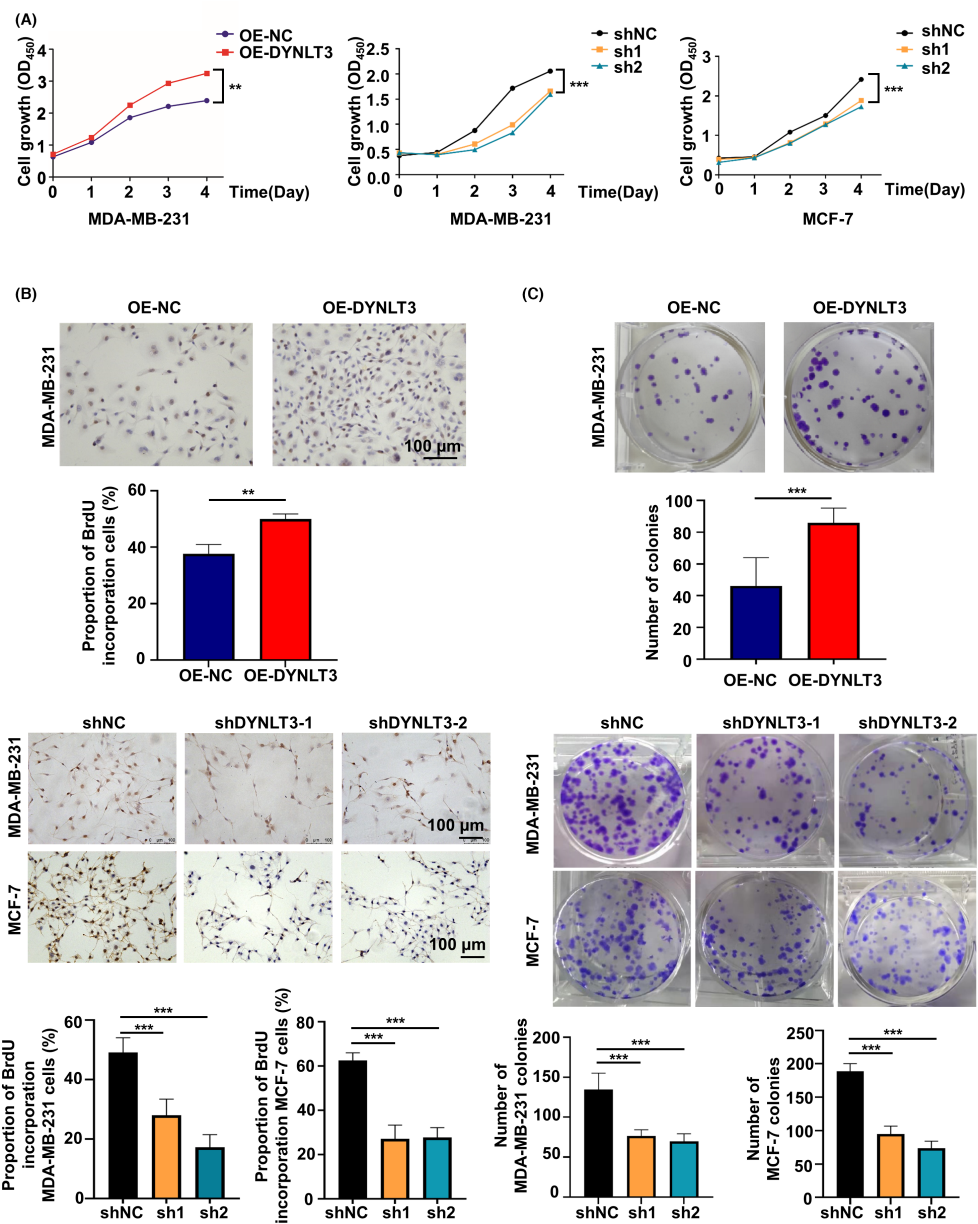
The effect of *DYNLT3* on cell viability and proliferation of breast cancer. (A) The cell viability was detected by CCK‐8 assay in *DYNLT3*‐knockdown and ‐overexpressed cells. (B) The stained cells with BrdU incorporation were used to evaluate cell proliferation. (C) The number of colonies formation was used to evaluate cell proliferation. **p < 0.01, ***p < 0.001.

First, cell viability was detected by the CCK‐8 assay. As shown in Figure 2A, the OD value curves suggested that overexpression of *DYNLT3* increased the viability of MDA‐MB‐231 cells, while *DYNLT3* knockdown decreased the cell viability in both MDA‐MB‐231 and MCF‐7 cells. Furthermore, compared to the corresponding control group, the overexpression of *DYNLT3* enhanced BrdU incorporation rate in MDA‐MB‐231 cells, which was about 50%, whereas sh*DYNLT3* groups decreased BrdU incorporation rate in MDA‐MB‐231 and MCF‐7 cells (Figure 2B). Similarly, clonal formation assay displayed that upregulation of *DYNLT3* promoted the number of cell colonies, which was 86, while downregulation of *DYNLT3* just attenuated this effect (Figure 2C). These results showed that *DYNLT3* could enhanced the proliferative ability of breast cancer cells.

### 3.3 DYNLT3 attenuates apoptosis of breast cancer cells

**FIGURE 3:**
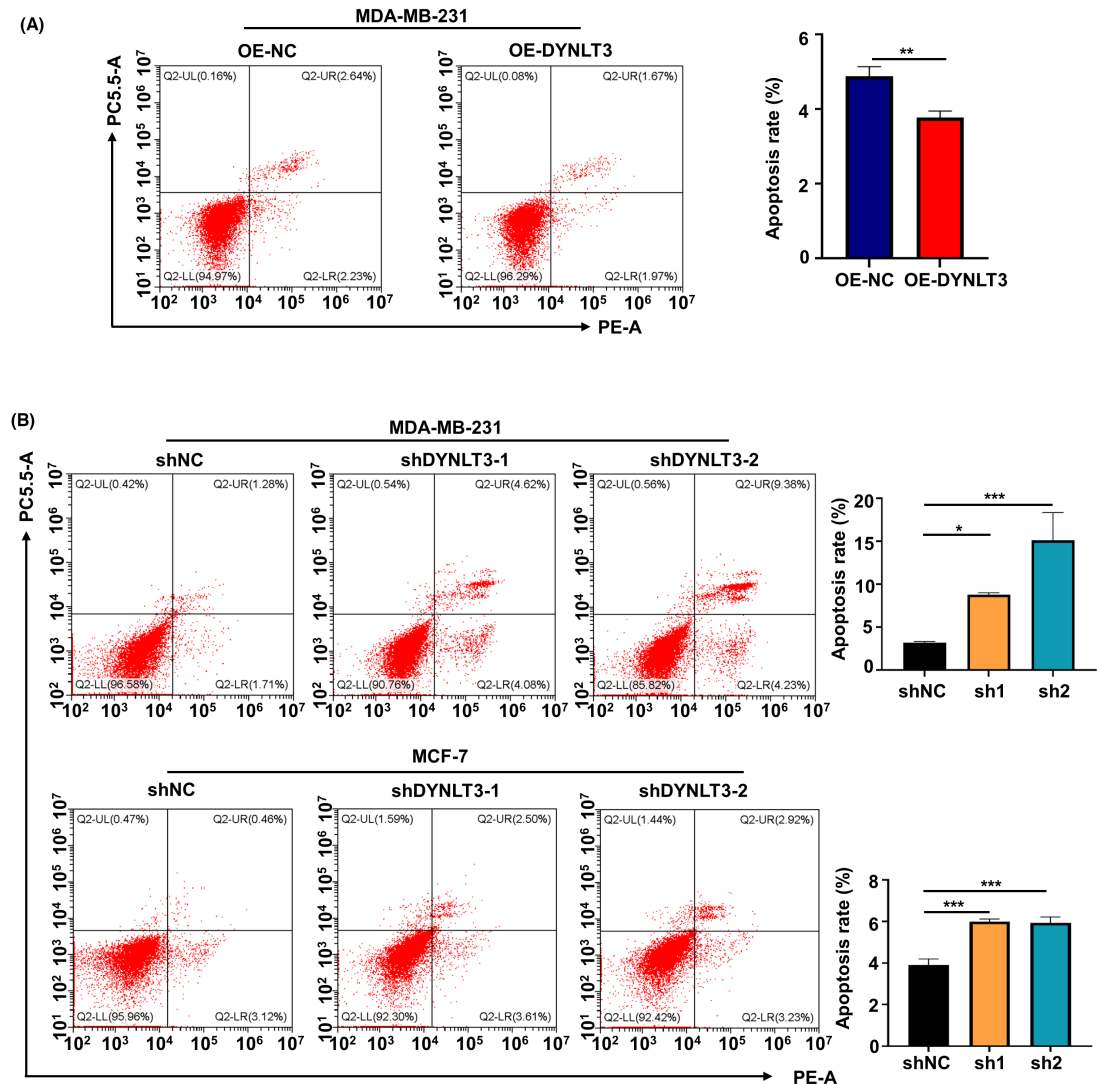
The effect of *DYNLT3* on the apoptosis of breast cancer cells. (A) The effect of *DYNLT3* overexpression on apoptosis of MDA‐MB‐231 cells. (B) The effect of *DYNLT3* knockdown on apoptosis of MDA‐MB‐231 cells and MCF‐7 cells. *p < 0.05, **p < 0.01, ***p < 0.001.

The role of *DYNLT3* on cell apoptosis was explored by the Annexin V‐PE/7AAD staining method. Experimental results found that the apoptosis rate of MDA‐MB‐231 cells was significantly reduced after *DYNLT3* overexpression (Figure 3A), which was about 3.7%. The apoptosis rate of *DYNLT3*‐knockdown cells was 8.7% and 13.61% in MDA‐MB‐231 cells, while was over 5% in MCF‐7, which was greatly higher in than that of the control groups (Figure 3B).

### 3.4 Expression of DYNLT3 promotes migration and invasion of breast cancer cells

**FIGURE 4:**
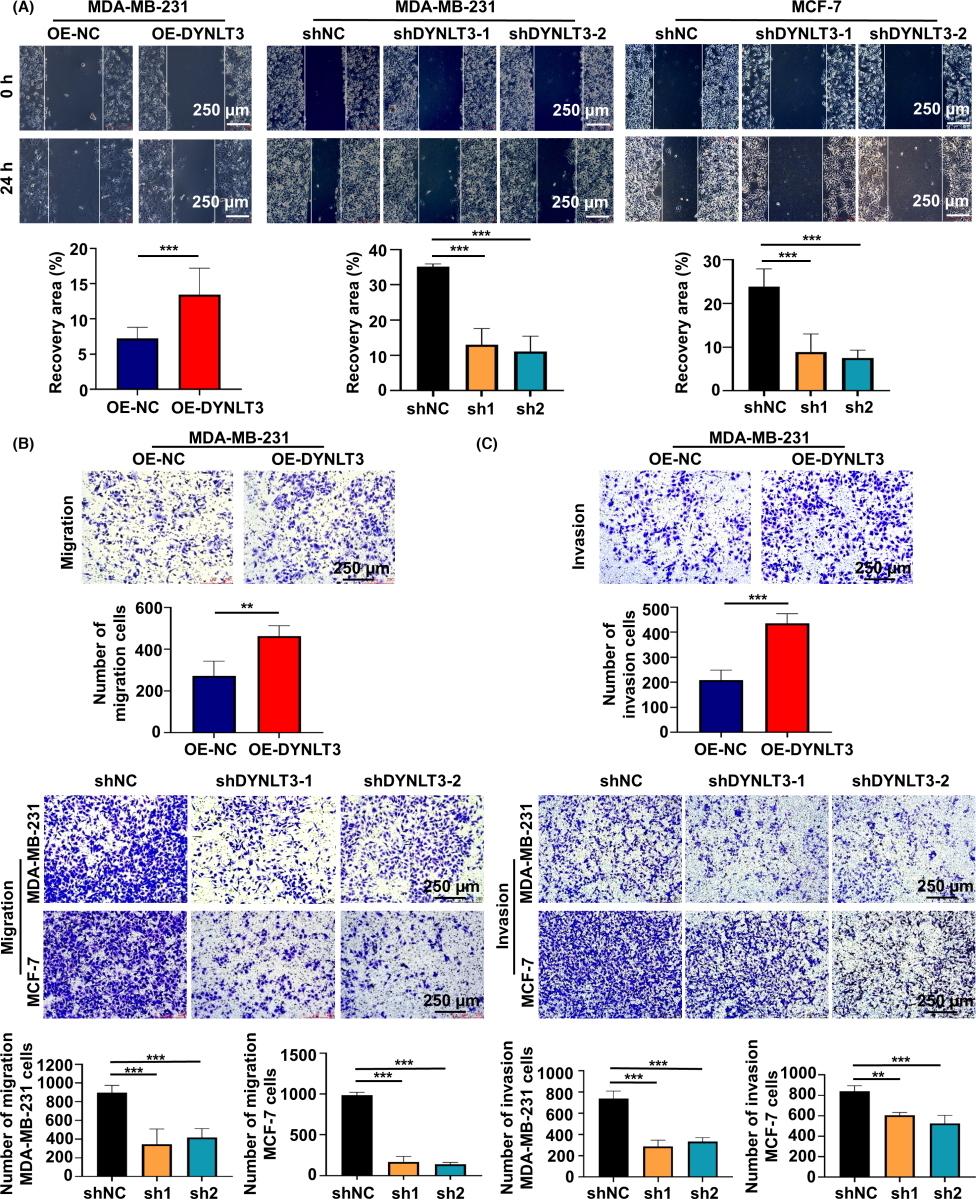
The effect of *DYNLT3* on breast cancer cell migration and invasion. (A) Wound healing experiment was used to detect the effect of *DYNLT3* expression on the migration of MDA‐MB‐231 cells and MCF‐7 cells. (B) Transwell method was used to detect the effect of *DYNLT3* expression on the migration of MDA‐MB‐231 cells and MCF‐7 cells. (C) Transwell assay was used to evaluate the effect of *DYNLT3* expression on the invasion of MDA‐MB‐231 cells and MCF‐7 cells. **p < 0.01, ***p < 0.001.

Wound healing experiments were performed to verify the effect of *DYNLT3* on cell migration and invasion. Figure 4A showed that the wound recovery capacity in *DYNLT3*‐overexpressed MDA‐MB‐231 cells was stronger. Expectedly, *DYNLT3* suppression weakened the wound recovery ability of MDA‐MB‐231 and MCF‐7 cells (Figure 4A). These results were further investigated by Transwell method. Upregulation of the *DYNLT3* expression improved the number of migratory MDA‐MB‐231 cells, which was 463, whereas silence of *DYNLT3* expression in MDA‐MB‐231 and MCF‐7 cells inhibited the number of migratory cells (Figure 4B). In addition, Transwell chambers with Matrigel were further utilized to assess the capacity of cell invasion. The overexpression of *DYNLT3* enhanced the cell invasion, there were 435 invasive cells in *DYNLT3*‐overexpressed MDA‐MB‐231 cells, while the ability was attenuated both in MDA‐MB‐231 cells and MCF‐7 cells after *DYNLT3* knockdown (Figure 4C).

### 3.5 DYNLT3 induces EMT in breast cancer cells

**FIGURE 5:**
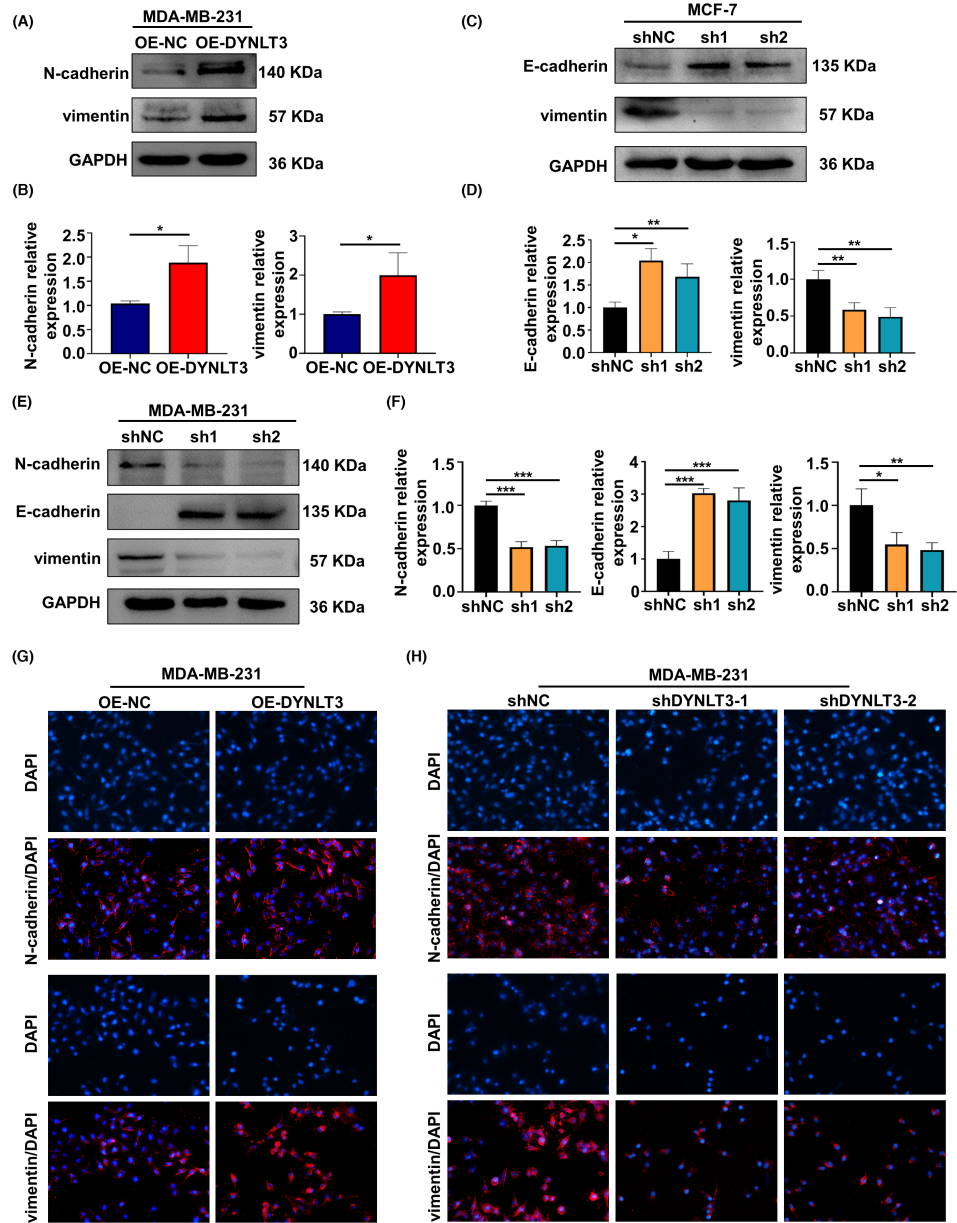
*DYNLT3* regulated the expression of EMT markers in breast cancer cells. (A) The expression level of N‐cadherin and vimentin protein in *DYNLT3*‐overexpressed MDA‐MB‐231 cells. (The N‐cadherin blots was detected from parallel experiments of the loading controls and vimentin by the same samples) (B) The quantitative analysis of (A). (C) The expression level of E‐cadherin and vimentin protein in *DYNLT3*‐knockdown MCF‐7 cells. (The E‐cadherin blots was detected from parallel experiments of the loading controls and vimentin by the same samples) (D) The quantitative analysis of (C). (E) The expression level of N‐cadherin, E‐cadherin and vimentin protein in *DYNLT3*‐knockdown MDA‐MB‐231 cells. (The N‐cadherin and vimentin blots were detected from parallel experiments of the loading controls and E‐cadherin by the same samples) (F) The quantitative analysis of (E). (G) Fluorescence images of overexpression *DYLNT3* cells to evaluate the N‐cadherin and vimentin expression. (H) Fluorescence images of *DYNLT3*‐knockdown cells to evaluate the N‐cadherin and vimentin expression. *p < 0.05, **p < 0.01, ***p < 0.001.

Furthermore, the expression of EMT markers in breast cancer cells was measured. Western blotting analysis showed N‐cadherin and vimentin were elevated in *DYNLT3‐*overexpressed MDA‐MB‐231 cells (Figure 5A,B). Besides, *DYNLT3* knockdown decreased the level of vimentin expression and induced E‐cadherin expression in MCF‐7 cells (Figure 5C,D). The Figure 5E,F also showed that knockdown *DYNLT3* not only downregulated the expression of N‐cadherin and vimentin but also upregulated the E‐cadherin in MDA‐MB‐231 cells. Moreover, the *DYNLT3*‐overexpressed MCF‐7 cells were constructed to investigate the change of E‐cadherin and vimentin expression. As shown in Figure S1A,B, the level of E‐cadherin expression was decreased and vimentin expression was increased in *DYNLT3*‐overexpressed MCF‐7 cells.

The IF assay also showed that N‐cadherin and vimentin were upregulated in *DYNLT3*‐overexpressed cells (Figures 5G and Figure S2A), while downregulated in *DYNLT3*‐knockdown cells (Figures 5H and Figure S2B).

### 3.6 Knockdown of the DYNLT3 expression reduces breast cancer growth in vivo

**FIGURE 6:**
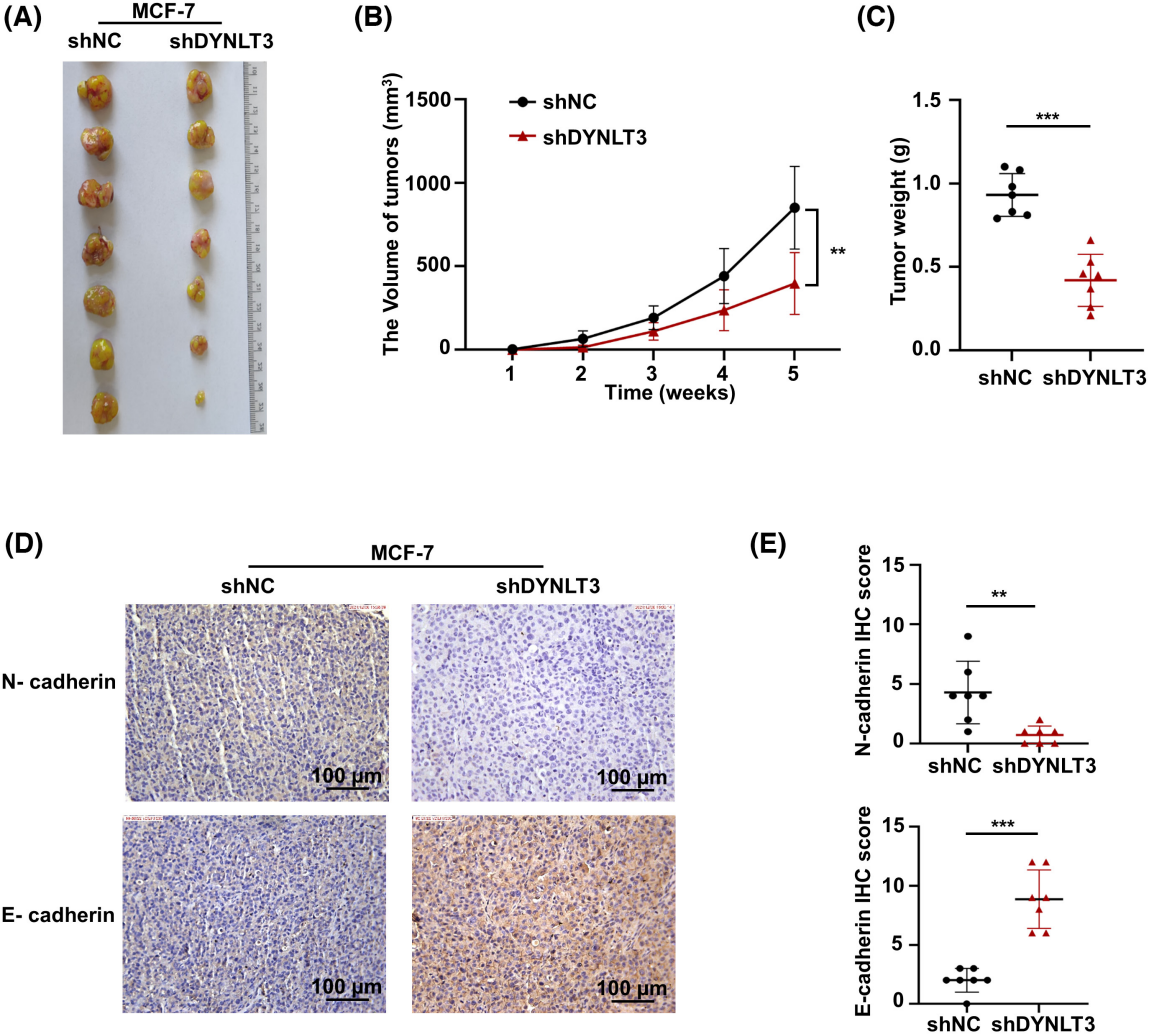
The effect of *DYNLT3* on breast cancer growth in vivo. The subcutaneous xenograft tumor model was formed by MCF‐7 cells knockdown of *DYNLT3* expression. (A) The xenograft tumors of nude mice were obtained at 5 weeks after injecting the MCF‐7 cells knockdown of *DYNLT3*. (B) The volume of tumors in the control group and *DYNLT3* knockdown group was detected to evaluate the tumor growth rate. (C) The weight of tumors was evaluated between the control group and *DYNLT3* knockdown group. (D) The IHC staining of N‐cadherin and E‐cadherin in xenograft tumor. (E) The IHC scores of N‐cadherin and E‐cadherin expression in tumor tissues. **p < 0.01, ***p < 0.001.

To further explore the effect of *DYNLT3* on the growth of breast cancer in vivo, the *DYNLT3*‐knockdown and control xenograft tumor models were established by subcutaneous injecting the corresponding MCF‐7 cancer cells in nude mice, respectively. Consistent with the in vitro results, the tumor growth rate was reduced and the volume of tumors was smaller after *DYNLT3* knockdown (Figures 6A,B). Furthermore, Figure 6C revealed that the tumor weight was significantly lighter in the *DYNLT3*‐knockdown group than that in the control group. Notably, compared with the control group, lower N‐cadherin and higher E‐cadherin expression were detected in tumor tissues from sh*DYNLT3* mice by IHC staining (Figures 6D,E).

## 4 DISCUSSION

Age‐related transcriptomic changes could drive breast cancer progression.^24^ The incidence and mortality of breast cancer increase with advancing age.^12^ *DYNLT3* has been identified as a gene associated with age of patients.^24^ In the present study, we found that *DYNLT3* expression was upregulated in breast cancer tissues and cells. *DYNLT3* overexpression promoted cell proliferation, EMT‐mediated migration and invasion, and inhibited apoptosis, while knockdown of *DYNLT3* exhibited the opposite functional biological role. Besides, the tumor formation of breast cancer cells in nude mice was inhibited by reducing *DYNLT3* expression.

*DYNLT3* belongs to the dynein light chain Tctex (*DYNLT*) family and is confirmed as a subunit common to dynein‐1 and dynein‐2.^25^ The oncogenic role of the *DYNLT* gene family in human cancer has already been demonstrated in several studies.^26^, ^27^ *DYNLT3* affect carcinogenesis might be related to the tumor types, exerting its oncogenic or tumor‐suppressive role in a tissue‐specific manner.^20^, ^23^

*DYNLT3* was identified as an age‐associated gene.^24^ As a result, the present research aimed to discover the potential mechanism of *DYNLT3* on breast cancer cell growth and development. The expression of *DYNLT3* was found to be highest in breast cancer tissues by IHC staining, and higher in breast fibroids than in normal tissues. Consistent with our study, several studies demonstrated that *DYNLT3* was upregulated in salivary gland adenoid cystic carcinoma, ovarian cancer and breast cancer.^19^, ^21^, ^24^

The loss‐ and gain‐of‐function experiments was performed to explore the character of *DYNLT3* in breast cancer. *DYNLT3* knockdown inhibited cell viability. The number of colonies formations and cancer cells with BrdU incorporation was suppressed when *DYNLT3* expression was silenced, while overexpression of *DYNLT3* improved cell viability, and the number of colonies formation and BrdU incorporation cells were increased. Sustaining cell proliferation is one of the phenotypes of malignancy, *DYNLT3* upregulation promoted proliferative ability of breast cancer cells. Animal experiments further confirmed that silence of *DYNLT3* attenuated breast cancer growth in vivo. *DYNLT3* knockdown could reduce the growth rate of xenograft tumors. The size and weight of tumors removed from sh*DYNLT3* nude mice groups were significantly reduced, which was consistent with experiments in vitro. Additionally, apoptosis can effectively eliminate damaged or mutated cells, which is a programmed cell death process. It is crucial for regulating organismal homeostasis during development and aging, and dysregulation of various apoptotic components can lead to the tumorigenesis. Our results showed that the apoptosis rate was reduced in *DYNLT3*‐overexpressed MDA‐MB‐231 breast cancer cells, and reversed in *DYNLT3*‐knockdown MDA‐MB‐231 and MCF‐7 breast cancer cells. These results collectively suggested that *DYNLT3* might maintain the survival of breast cancer cells by enhanced proliferation and reduce apoptosis, further promoting the occurrence and progression of breast cancer.

Scratch and Transwell assays showed an increment of wound healing percentage, and migratory and invasive abilities in *DYNLT3* overexpressed MDA‐MB‐231 cells. Knockdown of *DYNLT3* performed the opposite effect in MDA‐MB‐231 and MCF‐7 cells. Cancer metastasis is a multistep and complex process,^28^ which is one of the malignant biological characteristics of tumors and also a crucial factor for the prognosis of cancer.^7^ The process by which epithelial cells gradually acquire mesenchymal phenotypes is defined as EMT.^29^ The process of EMT is a dynamic transition and a key driver of cancer cell migration and invasion, which act as a vital regulated role in tumor metastasis and chemotherapy drug resistance.^10^, ^30^ A hallmark feature of EMT was the loss of epithelial cell integrity, which results in a decline in adhesion junctions that maintain interaction between epithelial cells. E‐cadherin, N‐cadherin, and vimentin can be used as markers of EMT.^31^, ^32^ Vimentin, a pivotal component of the cytoskeletal protein, is remodeled and upregulated during EMT, resulting in tumor cell survival and migration.^33^ The migratory and invasive capacities were regulated in breast cancer cells with *DYNLT3* expression. Furthermore, the indicators of the EMT process in breast cancer cells were altered by the level of *DYNLT3*. The overexpression of *DYNLT3* increased the expression of N‐cadherin and vimentin, whereas *DYNLT3* knockdown increased the expression of E‐cadherin while decreasing the expression of N‐cadherin and vimentin. Conventionally, MDA‐MB‐231 are characterized by the absence of E‐cadherin, however, *DYNLT3* knockdown induced the expression of E‐cadherin in MDA‐MB‐231 cells, which maintained the adhesion between tumor cells and inhibited migration. In animal experiments, higher expressions of E‐cadherin and lower expressions of N‐cadherin were found in tumor tissues from sh*DYNLT3* nude mice than in the control group. Consistent with our experimental findings, Zhu et al.^20^ explored that *DYNLT3* enhanced the migratory and invasive abilities of ovarian cancer. Zhang et al.^23^ also found that *DYNLT3* regulates EMT‐related proteins in cervical cancer. The EMT process in carcinogenesis is extremely complex, serving a pleiotropic function in the processes of cancer occurrence and metastasis.^34^ A decline of E‐cadherin and a rise of N‐cadherin induced EMT in breast cancer cells and promoted stemness, which is beneficial for driving proliferation potential.^35^ Ramirez et al.^36^ demonstrated that crosstalk occurred between E‐cadherin and epidermal growth factor receptors, and E‐cadherin could also be involved in regulation the of tumor growth. Wang et al.^37^ found that the absence of E‐cadherin enhanced the proliferative capacity of head and neck carcinoma via epidermal growth factor receptor transcriptional regulation.

Our results demonstrated that age‐associated gene *DYNLT3* may function as a tumor promoter in breast cancer by controlling the EMT process. This study provides an experimental basis for understanding and identifying the relationship between aging and tumorigenesis of breast cancer, which may define new opinions for the development of novel regimens for age‐related breast cancer treatment in the future. However, there are still some limitations in this current research. More clinical samples need to be collected to analyze the correlation between *DYNLT3* and hormone receptors expression pattern. The specific mechanism of DYNLT3 regulating EMT‐related factors to promote the proliferation and metastasis of breast cancer will be explored in our future research. In addition, the effect of using *DYNLT3* inhibitors on breast cancer recurrence and metastasis needs to be explored both in vitro and in vivo. Whether the expression of *DYNLT3* improves the efficacy of other chemotherapeutic agents for breast cancer, especially TNBC, remains to be further investigated.

## 5 CONCLUSION

In brief, age‐associated gene *DYNLT3* acts as a pivotal factor in the breast tumorigenesis and metastasis. Silence of *DYNLT3* expression inhibited cell viability, migration, and invasion, and enhanced the proportion of apoptotic cells, while the proliferative, migratory and invasive abilities in *DYNLT3‐*overexpressed breast cancer cells were improved. Additionally, the expression of *DYNLT3* is involved in the process of EMT with regulation of E‐cadherin, N‐cadherin, and vimentin expression. Further identification of the specific molecular mechanisms and signaling pathways by which *DYNLT3* regulates EMT may provide advanced insights into the novel treatment of age‐related breast cancer.

## AUTHOR CONTRIBUTIONS

**Han Wang:** Investigation (lead); methodology (equal); visualization (equal); writing – original draft (supporting). **Xin Chen:** Investigation (supporting); methodology (equal); visualization (equal); writing – original draft (lead). **Yanshan Jin:** Validation (equal); visualization (supporting); writing – original draft (supporting). **Tingxian Liu:** Validation (equal); visualization (supporting); writing – original draft (supporting). **Yizuo Song:** Investigation (supporting); validation (supporting); writing – original draft (supporting). **Xuejie Zhu:** Conceptualization (equal); project administration (equal); writing – original draft (supporting). **Xueqiong Zhu:** Conceptualization (equal); project administration (equal); supervision (equal); writing – original draft (supporting).

## CONFLICT OF INTEREST STATEMENT

The authors declare that there are no conflicts of interest regarding the publication of this paper.

## ETHICS STATEMENT

The experiments in this research were performed in line with the ethics committee of the Second Affiliated Hospital of Wenzhou Medical University (KY‐2017‐43), and the participants included in this experiment signed preoperative informed consent.

## Supporting information


**Figure S1.** **Figure S2**.

## Data Availability

The data in this study are available from the corresponding author on reasonable request.
