# Supplementary material for: The role of DYNLT3 in breast cancer proliferation, migration, and invasion via epithelial‐to‐mesenchymal transition
Source: Cancer Med. 2023 Jun 1;12(14):15289–303. doi: 10.1002/cam4.6173 (PMC10417059; doi:10.1002/cam4.6173)
Supplement: Supplementary file 1 — Figure S1. Figure S2. [file CAM4-12-15289-s001.docx]

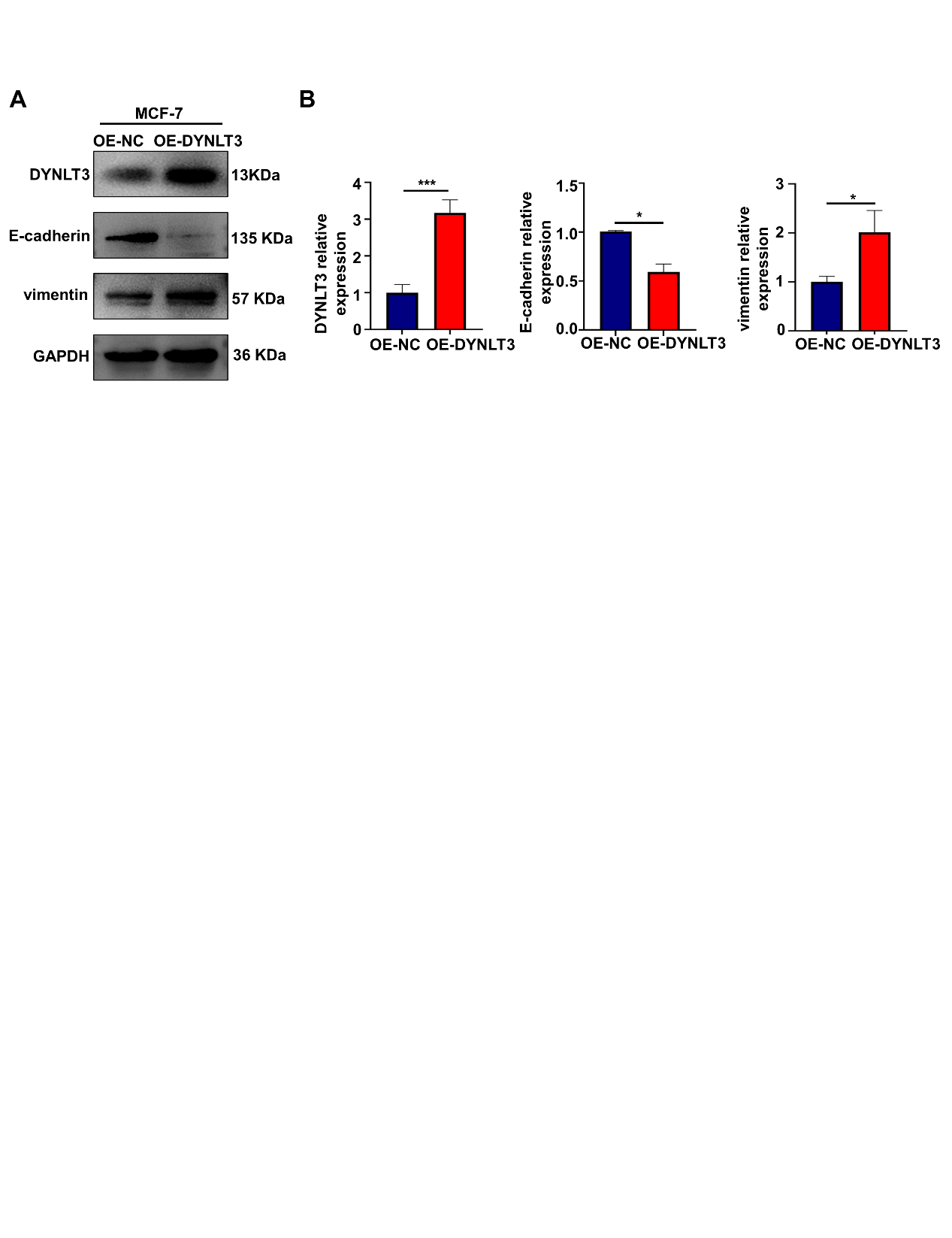


**Figure S1** The role of *DYNLT3* overexpression on the regulation of E-cadherin and vimentin expression in MCF-7 breast cancer cells. (A) The expression level of *DYNLT3*, E-cadherin and vimentin protein in *DYNLT3*-overexpressed MCF-7 cell. (B) The quantitative analysis of (A). ^*^*P*<0.05, ^***^*P*<0.001


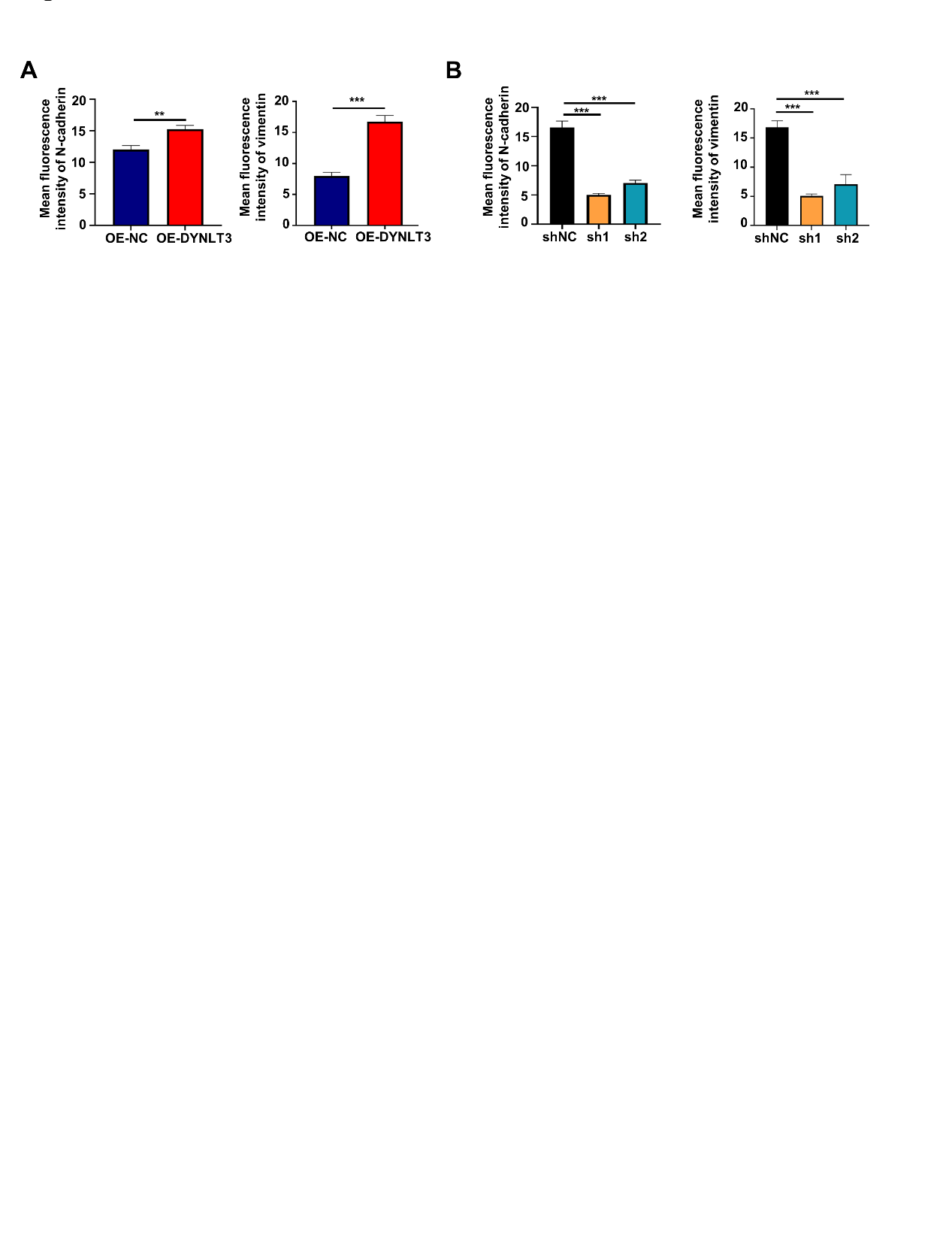


**Figure S2** The relative fluorescence images of *DYNLT3*-knockdown cell to evaluate the N-cadherin and vimentin expression. (A) The quantitative analysis of N-cadherin and vimentin fluorescence expression in MDA-MB-231 cells with *DYNLT3* overexpression. (B) The quantitative analysis of N-cadherin and vimentin fluorescence expression in MDA-MB-231 cells with *DYNLT3* knockdown. ^**^*P*<0.01, ^***^*P*<0.001
